# Interleukin-33/suppression of tumorigenicity 2 (IL-33/ST2) axis in idiopathic inflammatory myopathies and its association with laboratory and clinical parameters: a pilot study

**DOI:** 10.1007/s00296-020-04554-z

**Published:** 2020-03-28

**Authors:** Aleksandra Opinc, Joanna Sarnik, Olga Brzezińska, Marcin Makowski, Anna Lewandowska-Polak, Joanna Makowska

**Affiliations:** 1 Department of Rheumatology, Medical University of Lodz, ul. Pieniny 30, 92-115 Łódź, Poland; 2 Departament of Intensive Care, Cardiology, Medical University of Lodz, ul Pomorska 251, 92-213 Łódź, Poland

**Keywords:** Idiopathic inflammatory myopathy, ST2, IL-33, Myositis

## Abstract

Idiopathic inflammatory myopathies (IIM) are rare connective tissue diseases, which can lead to internal organ involvement. IL-33/ST2 pathway is involved in the pathogenesis of numerous diseases including autoimmune disorders. IL-33 fulfils cardioprotective function, while soluble ST2 (sST2) is a decoy receptor that reduces protective impact of IL-33. The aim of the study was to evaluate the concentrations of sST2 and IL-33 in sera of patients with IIM and evaluate its associations with the clinical course of the disease. Patients with IIM as well as age- and sex-matched healthy controls were recruited. Concentrations of sST2 and IL-33 were assessed with ELISA in sera of both patients and controls. Patients were asked to fill in the questionnaires concerning clinical symptoms and physical functioning. Concentrations of sST2 and IL-33 were correlated with the results of laboratory tests and clinical symptoms. Concentrations of sST2 were significantly higher in IIM group than in healthy subjects (median sST2 in IIM 26.51 vs in healthy controls 21.39; *p* = 0.03). In the majority of patients, IL-33 concentrations did not exceed the detection limit. Anti-SRP-positive patients presented significantly higher concentrations of sST2 as compared to anti-SRP-negative patients (*p* = 0.04). In patients with anti-Ro52 antibodies, sST2 concentrations were significantly lower than in anti-Ro52-negative patients (*p* = 0.02). Concentrations of sST2 correlated with the degree of disability evaluated with Health Assessment Questionnaire. sST2 is increased in patients with IIM and its concentration correlates with the degree of disability. In patients with anti-SRP antibodies, levels of sST2 are exceptionally high.

**Electronic supplementary material:**

The online version of this article (10.1007/s00296-020-04554-z) contains supplementary material, which is available to authorized users.

## Introduction

IIM is usually associated with muscle inflammation leading to progressive weakness; however, also the skin and internal organs may be affected [1, 2]. The role of IL-33/ST2 (interleukin-33/suppression of tumorigenicity 2) pathway has been studied recently in various diseases including infectious, autoimmune, metabolic and degenerative conditions [3]. IL-33/ST2 signalling participates in inflammatory resolution, not only leading to tissue reparation but also provoking fibrosis of potentially any internal organ [3]. After muscular injury, IL-33 regulates the accumulation of regulatory T cells [3]. Interaction of sST2 and macrophages can down-regulate pro-inflammatory cytokines [4]. IL-33 fulfils cardioprotective function, especially in response to myocardial stress, imposing an anti-hypertrophic effect due to blocking the influence of angiotensin II and phenylephrine on cardiac muscle, reducing fibrosis and enhancing cardiomyocytes survival [5, 6]. sST2, being a decoy a receptor for IL-33, suppress its pleiotropic impact on both innate and adaptive responses and has emerged as a novel prognostic biomarker of cardiac dysfunction [3].

The aim of the study was to evaluate the concentrations of IL-33 and sST2 in sera of patients with IIM, as well as its association with clinical manifestations, laboratory tests and autoantibodies profile.

## Patients and methods

Sixteen (16) consecutive patients with IIM (polymyositis, dermatomyositis, antisynthetase syndrome, myositis with overlapping scleroderma), being hospitalized in the Department of Rheumatology between 2018 and 2019 and agreed to participate, were recruited. Diagnosis were based on the Peter and Bohan criteria [7, 8]. All the patients included in the study group met also the EULAR/ACR classification criteria for adult and juvenile idiopathic inflammatory myopathies and their major subgroups—seven patients had define IIM, nine probable IIM [9]. Seventeen (17) age- and gender-matched healthy subjects were enrolled as the control group. Exclusion criteria for the control group included any autoimmune, musculoskeletal or cardiovascular diseases.

9 ml of peripheral blood were collected from both patients and healthy controls, serum samples were stored for subsequent IL-33 and sST2 analysis. Levels of IL-33 and sST2 were measured by commercially available enzyme-linked immunosorbent assays (ELISA) in serum samples. For sST2 estimation, we used The Human ST2 ELISA Kit (Biorbyt Ltd., Cambridge, United Kingdom, product number: orb219434) and for the measurement of IL-33 Human IL33 ELISA Kit (Biorbyt Ltd., Cambridge, United Kingdom, product number: orb390944). For sST2 analysis, the samples were diluted 3:100 (serum:diluent). No samples dilutions were made for IL-33 measurement. Assay procedures were followed according to the manuals provided by the manufacturer (protocols with an overnight incubation at 4 °C). Optical density was measured by plate reader at 450 nm and concentrations were automatically interpolated form the standard curve. The concentration of sST2 obtained in diluted-sera were multiplied by the dilution factor to determine the concentrations in the non-diluted samples. All the results concerning sST2 in the result section are presented after multiplication by the dilution factor. During hospitalization, patients were asked to fill in the questionnaire prepared by the authors in Polish language (native language of the participants), concerning symptoms and concomitant diseases. Degree of myalgia, muscle weakness, fatigue and tolerance of physical activity were self-assessed by the patients in 0–10 scale, where 0 indicated no presence of evaluated feature and 10 the highest possible intensity that impairs daily activities. Patients were also asked to assess their physical functioning by filling in the Short 2-page Health Assessment Questionnaire, referred as the Health Assessment Questionnaire-Disability Index (HAQ-DI, Polish translation). Standard and Alternative Disability Indexes (SDI, ADI) were counted according to the instructions contained in The Health Assessment Questionnaire (HAQ) disability index (DI) of the clinical health assessment questionnaire (version 96.4) [10]. Results of laboratory tests such as complete blood count, erythrocyte sedimentation rate (ESR), C-reactive protein (CRP), creatine kinase (CK) and its muscle-brain isoform (CK-MB), myoglobin, N-terminal pro-brain peptide (NT-proBNP), alanine aminotransferase (ALT) and aspartate aminotransferase (AST) were obtained retrospectively from medical documentation if performed during the same hospitalization as serum samples collection. Titres and types of antinuclear antibodies (ANA) were also obtained retrospectively. Cardiac troponin T and I were assessed in the blood samples collected from the patients.

Data were analysed with STATISTICA 13.1 software. The normality of data was tested by Shapiro–Wilk test. Concentration of sST2 and IL-33 was compared between patients with IIM and healthy controls with *U* Mann–Whitney test. Associations between clinical symptoms, comorbidities or ANA subtypes with sST2 and IL-33 concentrations were evaluated with *U* Mann–Whitney tests (for symptoms and comorbidities only if they were present in at least four patients). Linear regressions were performed to evaluate the influence of the laboratory parameters, myalgia, muscle weakness, fatigue, tolerance of physical activity and degree of disability on sST2 and IL-33 levels.

The procedures followed were in accordance with the ethical standards and were approved by the responsible bioethical committee on human experimentation (Bioethical Committee of the Medical University of Lodz, Poland, date of approval: 15.05.2018, reference number: RNN/173/18/KE). All patients gave written, informed consent for participation in the study.

## Results

Clinical characteristics of the patients enrolled for the study as well results of laboratory tests are presented in Table 1. Half of the patients declared exertional dyspnoea or episodes of chest pounding/irregular heartbeat occurring currently or in the past after the diagnosis of IIM. Most common comorbidities in patients with IIM included hypertension, thyroid disorders, interstitial lung disease and hypercholesterolemia/atherosclerosis. 10 patients filled in the Short 2-page Health Assessment Questionnaire. According to SDI, 30% fulfilled the criteria of mild-to-moderate disability, 40% of moderate-to-severe disability and 30% of severe-to-very severe disability.

**Table 1:** Clinical characteristics of the patients and healthy controls recruited for the study

		IIM *N* = 16 [median; min–max]	Control group *N* = 17 [median; min–max]	*p*
Demographical data				
Gender		*F* = 8; *M* = 8	*F* = 8; *M* = 9	> 0.05
Age (years)		59.50; 25–76	50.00; 25–65	> 0.05
Disease duration (months)		9.5; 1–52	–	–
Clinical symptoms present at any time after diagnosis [*n* (%)]				
Arthralgia		7 (43.75)	–	–
Dysphagia		6 (37.5)	–	–
Dysphonia		7 (43.75)	–	––
Erythema		5 (31.25)	–	–
Gottron papules/sign		3 (18.75)	–	–
Raynaud phenomenon		2 (12.5)	–	–
Mechanic’s hands		3 (18.75)	–	–
Dyspnoea at rest		4 (25.00)	–	–
Exertional dyspnoea		8 (50.00)	–	–
Chest pain		2 (12.5)	–	–
Irregular heartbeat, chest pounding		8 (50.00)	–	–
Dry cough		3 (18.75)	–	–
Productive cough		3 (18.75)	–	–
Fever		2 (12.5)	–	–
VAS 0–10				
Myalgia		6.25; 0–10 (*N* = 14)	–	–
Muscle weakness		6.50; 0–10 (*N* = 14)	–	–
Fatigue		5.00; 0–9 (*N* = 14)	–	–
Tolerance of physical activity		7.00; 3–10 (*N* = 13)	–	–
Health Assessment Questionnaire				
ADI		1.06; 0.25–3.00 (*N* = 10)	–	–
SDI		1.25; 0.25–3.00 (*N* = 10)	–	–
Most common comorbidities [*n* (%)]				
Hypertension		9 (56.25)	–	–
Thyroid disorders		7 (43.75)	–	–
Interstitial lung disease/unspecified interstitial lesions		7 (43.75)	–	–
Hypercholesterolemia, atherosclerosis		5 (31.25)	–	–
Arrhythmia		3 (18.75)	–	–

Laboratory tests				
Test	Norm			
WBC (× 10^3^/µl)	4.00–11.00	8.18; 2.56–18.74 (*N* = 15)	–	–
RBC (× 10^6^/µl)	4.20–6.10 (*M*) 3.80–5.40 (*F*)	4.45; 3.71–5.68 (*N* = 15)	–	–
Hb (g/dl)	14.0–18.0 (*M*) 3.80–5.40 (*F*)	13.60; 11.50–16.10 (*N* = 15)	–	–
PLT (× 10^3^/µl)	150–400	282.00; 195.00–342.00 (*N* = 15)	–	–
ESR (mm/1 h)	< 20	14.00; 4.00–38.00 (*N* = 15)	–	–
CRP (mg/l)	0.0–6.0	2.40; 0.40–23.70 (*N* = 15)	–	–
CK (U/l)	0.0–171.0	155.00; 20.00–25,463.00 (*N* = 15)	–	–
CK-MB (ng/ml)	< 4.94	6.01; 2.39–252.20 (*N* = 7)	–	–
Troponin T (ng/l)	< 14	65.56; 9.98–2061.00 (*N* = 15)	–	–
Troponin I (ng/l)	< 19	12.70; 1.80–451.10 (*N* = 15)		
AST (U/l)	0.0–35.0	31.70; 13.60–671.50 (*N* = 15)	–	–
ALT (U/l)	0.0–45.0	38.50; 7.50–577.60 (*N* = 15)	–	–
Myoglobin (µg/l)	23.0–72.0	142.00; 21.10–3000.00 (*N* = 12)	–	–
NT-pro BNP (pg/ml)	< 125.0	329.00; 34.50–1412.00 (*N* = 6)	–	–
Subtypes of ANA [*n* (%)]				
Jo-1		8 (50.00)	–	–
Ro-52		10 (62.50)	–	–
SRP		3 (18.75)	–	–
Pm-Scl		4 (25.00)	–	–
AMA-M2		3 (18.75)	–	–
24 h ECG holter monitoring [*n* (%)]				
> 2000 supraventricular extrasystoles		2 (22.23) (*N* = 9)	–	–
> 200 ventricular extrasystoles		5 (55.56) (*N* = 9)	–	–
> 2000 ventricular extrasystoles		3 (33.34) (*N* = 9)	–	–
Soluble ST2 and IL-33 concentrations				
sST2 in serum sample (ng/ml)		26.51; 13.12–68.67	21.39; 15.24–32.40	< 0.05
IL-33 in serum sample (ng/ml)		0.4019; < 15.6–956.35	1.5404; < 15.6–1051.64 (*N* = 10)	> 0.05

*IIM* idiopathic inflammatory myopathy, *N* number of patients, *F* female, *M* male, *ANA* antinuclear antibodies, *VAS* visual analogue scale

All the 16 patients were ANA-positive, specific autoantibodies were detected in 15 patients (93.75%). 11 out of 16 patients included in the study group had myositis-specific autoantibodies, four patients had only myositis-associated autoantibodies. In one patient (ANA positive), no specific autoantibodies were detected. The most common subtype of ANA was anti-Ro52 in 62.50% of patients, followed by anti-Jo1 in 50% of cases, anti-PM-Scl in 25%, anti-SRP in 18.75% and AMA-M2 in 18.75% (Table 1). Single cases of anti-Ku, anti-HI and anti-centromere antibodies were detected.

Mean values of complete blood count, ESR and CRP in patients remained within normal limit, whereas levels of CK, CK-MB, myoglobin, NT-proBNP, AST and ALT were elevated (Table 1). Noteworthy, 87% out of 15 patients with IIM presented elevated levels of troponin T, whereas troponin I was increased only in 20% of them (Table 1).

Concentrations of sST2 in sera ranged from 13.12 to 68.67 ng/ml in patients with IIM and from 15.24 to 32.40 ng/ml in healthy controls. Concentrations of sST2 were significantly higher in IIM group than in healthy subjects (median sST2 in IIM 26.51 ng/ml vs median sST2 in healthy controls 21.39; *p* = 0.03). Concentrations of IL-33 in sera ranged from below the detection limit to 956.35 pg/ml. In half of the patients with IIM and half of the control group concentrations of IL-33 did not exceed the detection limit of 15.6 pg/ml. No significant difference was observed in serum concentrations of IL-33 between patients with IIM and healthy controls (respectively, median 0.4019; < 15.6 to 956.35 in IIM group vs median 1.5404; < 15.6 to 1051.64 in control group; *p* = 0.8).

**Fig. 1:**
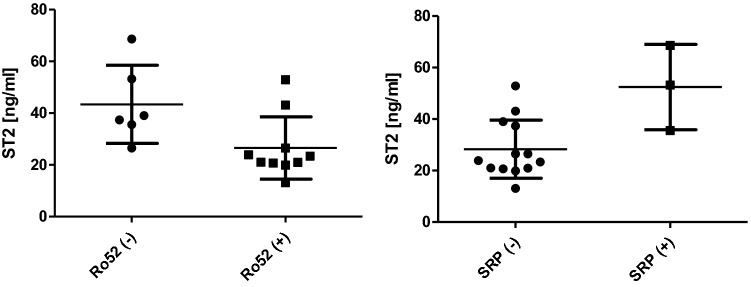
Comparison between soluble ST2 concentration in anti-Ro52-positive and anti-Ro52-negative patients as well as anti-SRP-positive and anti-SRP-negative patients

**Fig. 2:**
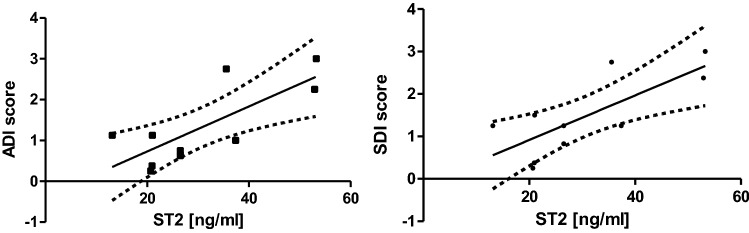
Positive correlations between ADI and soluble ST2 (*r* = 0.769; *p* = 0.01) and between SDI and soluble ST2 (*r* = 0.764; *p* = 0.01) in sera of IIM patients

No significant difference was observed in concentrations of sST2 and IL-33 between patients with and without dyspnoea, chest pounding/irregular heartbeat and remaining clinical symptoms such as arthralgia, dysphonia, dysphagia, erythema. Comorbidities, degree of myalgia, muscle weakness, fatigue and tolerance of physical activity were neither associated with significantly higher concentrations of sST2 or IL-33. Anti-SRP-positive patients presented significantly higher concentrations of sST2 as compared to anti-SRP-negative patients (Fig. 1, mean value of 52.49 ± 16.57 ng/ml in anti-SRP-positive patients vs mean value of 28.33 ± 11.32 ng/ml in anti-SRP-negative patients; *p* = 0.04). In contrast, in patients with anti-Ro52 antibodies, sST2 concentrations were significantly lower than in anti-Ro52-negative patients (Fig. 1, mean value of 26.54 ± 12.05 ng/ml in anti-Ro52-positive patients vs mean value of 43.40 ± 15.09 ng/ml in anti-Ro52-negative patients; *p* = 0.02). Both ADI and SDI scores correlated with the sST2 concentrations (Fig. 2, respectively, *r* = 0.769; *p* = 0.01 for ADI and *r* = 0.764; *p* = 0.01 for SDI). Serum levels of sST2 and IL-33 did not correlate with the results of laboratory tests.

## Discussion

To our knowledge, the role of IL-33/ST2 pathway in patients with IIM has been previously evaluated only in a single study [11]. Yuan et al. noted significantly higher levels of sST2 in dermatomyositis/polymyositis (DM/PM) patients as compared to healthy controls [11]. This observation remains in line with the results of our study, as we also obtained significantly higher concentrations of sST2 in patients with IIM than in the control group. Authors observed also a strong positive correlation between sST2 levels and global VAS score [11], which partially corresponds with our results, as we demonstrated a positive correlation between sST2 levels and degree of disability assessed with HAQ-DI. Contrary to our study, in the research conducted by Yuan et al., serum sST2 levels correlated with CRP, CK and LDH [11]. In the study by Yuan et al. concentrations of IL-33 in DM/PM patients were not significantly higher than in the control group [11]. This observation also corresponds with our results, yet it need to be highlighted than in our study half of the patients had IL-33 concentrations below the detection limit.

Half of the patients from our study group experienced palpitations and exertional dyspnoea. This corresponds with the results conducted by Diederichsen et al. on the larger study group, in which 57% of IIM patients complained of dyspnoea and 34% reported palpitations [12]. In our study, majority of patients with IIM presented elevated levels of troponin T, whereas troponin I was increased only in 20% of them. However, in patients with myositis coexistence of cardiac and muscular lesions may distort the results of laboratory tests. Elevated concentrations of cardiac troponin T (cTnT) and CK-MB may result not only from the injury of cardiomyocytes but also from regenerating skeletal myocytes [13–15]. High concentrations of cardiac cTnT might be associated with severe course of myopathy [16]. Despite those facts, it is recommended to start the assessment in clinical practice with cTnT and evaluate cardiac troponin I (cTnI) in those who present increased concentrations of cTnT [14]. sST2 is associated with increased risk of death, heart failure or ventricular arrhythmia, as it reduces protective impact of IL-33 on myocardium [6, 17]. Yet, in our study, no correlations were found between IL-33, sST2 and clinical symptoms indicating cardiovascular involvement or biochemical markers of cardiac injury such as CK-MB, troponin T, troponin I or NT-proBNP. High concentrations of cardiac troponin T, that we observed in our study, may, therefore, be the result of severe muscle involvement. A lager study group could enable to state more reliable conclusions.

We analysed in detail the association between sST2 concentrations and different subtypes of ANA. Yuan et al. displayed no correlation between sST2 and anti-Jo-1 and anti-Mi2 antibodies [11]. Similarly, in our study, anti-Jo-1 and concentrations of sST2 did not correlate. However, we observed higher sST2 concentrations in anti-SRP-positive patients, what to our knowledge has not been stated before. Signal Recognition Particle (SRP) regulates transport of proteins through the endoplasmic reticulum membrane [18]. Antibodies targeting this protein-RNA complex are associated with poor response to treatment and might have been related to cardiac complication. In a study on 12 anti-SRP-positive PM patients 25% of them presented arrhythmia [19]. Case of anti-SRP-positive patient with IIM, who developed inflammatory cardiomyopathy was described [20]. In other case report, anti-SRP-positive myopathy led to myopericarditis [21]. In our study, all the three patients with anti-SRP antibodies declared irregular heartbeat/chest pounding. Two out of three patients declared high intensity of myalgia and minimal tolerance of physical activity. One anti-SRP-positive patient declared no myalgia at all. Furthermore, two anti-SRP-positive patients shared also comorbidities—both of them were diagnosed also with hypertension, ischemic heart disease, arrhythmia and heart failure. However, as no correlations were observed between sST2 concentration and intensity of myalgia or tolerance of physical activity, as well as between patients with and without clinical symptoms or particular comorbidities, observed difference could be related more to the presence of anti-SRP antibodies itself. Recently, anti-mitochondrial (AMA-M2)-positive phenotype was suggested to be related with cardiac involvement in IIM [22, 23], yet in our study we did not observe any correlation between AMA-M2 and concentration of sST2. However, we observed an increased level of troponin I in 3 out of 15 patients—1 of these patients was anti-SRP-positive, 1 AMA-M2-positive and 1 anti-Ku-positive.

We presented for the first time the association between anti-Ro52 antibodies and sST2 levels in patients with IIM. Anti-Ro52 is described as the most common myositis-associated antibody in adults with myositis, what corresponds with our results [24]. Presence of anti-Ro antibodies in PM may be associated with conductive disturbances such as atrioventricular heart blocks and bundle branch blocks [25]. Anti-Ro52 antibodies are also associated with interstitial lung disease (ILD) and coexistence of anti-Jo-1 and anti-Ro52 with severe course of ILD [26]. In our study, 7 out of 16 patients had ILD and 85.71% of those with ILD were anti-Ro52 positive. The highest mRNA transcriptional content of sST2 was found in lungs, heart and brain tissue. Secretion of sST2 from myocytes and pneumoepithelial cells is enhanced by pro-inflammatory cytokines [4]. Surprisingly, in our study anti-Ro52-positive patients presented lower levels of sST2. Furthermore, sST2 concentrations in patients with ILD did not differ as compared to patients without ILD. Researchers have found that levels of sST2 mRNA transcription are higher in bronchial and alveolar epithelial cells as compared to lung fibroblasts, therefore, sST2 may be associated with the airway diseases rather than ILD [4].

The main limitation of our study, that could impact the results, is that study group was limited and highly heterogeneous as for the age of the patients, disease duration and disease activity, subtypes of antinuclear antibodies. Yet, IIM are rare disorders with the prevalence ranging from 2.4 to 33.8/100000 and our study was a single-centre observation with a short duration time [27]. Some of the subgroups of patients (for example anti-SRP-positive) consisted of only few patients, therefore, studies on larger number of patients are required to form reliable conclusions. Furthermore, results of laboratory tests as well as filled questionnaires (especially HAQ-DI) were not available for every patient enrolled for a study. Moreover, our study lack echocardiographic examination. According to literature, left ventricular diastolic dysfunction and echocardiographic lesions indicating diastolic impairment are frequently seen in patients with myositis [12, 28]. Therefore, we cannot exclude the possibility of missing features of cardiac involvement. A study on larger group with assessment of the echocardiographic lesions would be necessary to draw more definite conclusions.

## Conclusions

Soluble ST2 is increased in patients with IIM and its concentration correlates with degree of disability. No correlations were found between IL-33 and sST2 concentrations and clinical symptoms or biochemical tests. sST2 concentrations may be associated with autoantibody profile as in patients with anti-SRP antibodies levels of sST2 are exceptionally high.

### Related congress abstracts

1. Partial results of the study, concerning clinical symptoms, results of 24 hour ECG Holter monitoring and results of biochemical tests in some of the patients from the study group have been presented at the students’ conference: 57th Polish and 15th International Conference Juvenes Pro Medicina 2019, organized by The Students’ Scientific Association of the Medical University of Lodz, Poland, 24.05.2019-25.05.2019 in Lodz. Abstracts have been published in the Abstract Book (no DOI available): Opinc A (2019) Does heart matter in idiopathic inflammatory myopathies? Observations from a single-centre study, Abstract Book Juvenes Pro Medicina 24–25 May 2019, pp 191. ISBN 978-83-947627-2-8. https://jpm.umed.pl/pliki/abstractbook/Juvenes-Pro-Medicina-2019.pdf
2. The questionnaire prepared by the authors (English version) was previously used in another study. The results of this study have been presented as a poster on Annual European Congress of Rheumatology, EULAR 2019, Madrid, 12–15 June 2019: Opinc A, Brzezińska O, Makowska J. FRI0685 prevalence of cardiovascular symptoms in patients with idiopathic inflammatory myopathies—a questionnaire based study. Annals of the Rheumatic Diseases 2019;78:1041–1042. https://doi.org/10.1136/annrheumdis-2019-eular.7757

## Electronic supplementary material

Below is the link to the electronic supplementary material.

Supplementary file1 (DOCX 12 kb)
