# Supplementary material for: Interleukin-33/suppression of tumorigenicity 2 (IL-33/ST2) axis in idiopathic inflammatory myopathies and its association with laboratory and clinical parameters: a pilot study
Source: Rheumatol Int. 2020 Mar 28;40(7):1133–41. doi: 10.1007/s00296-020-04554-z (PMC7256085; doi:10.1007/s00296-020-04554-z)
Supplement: Supplementary file 1 — Supplementary file1 (DOCX 12 kb) [file 296_2020_4554_MOESM1_ESM.docx]

All procedures during the ST2 ELISA test have been performed in accordance to the manual provided by the producer with ELISA kit (https://www.biorbyt.com/pub/media/additional_documents/o/r/orb219434.pdf).
According to the manual „The user will decide sample dilution fold by crude estimation of protein amount in samples”. Therefore, in order to estimate the dilutions, we have conducted an initial experiment using maximum concentration of standard sample, sera from 2 randomly selected patients from the IIM group and 1 sample from the control group. For each sample, we performed the ELISA test for several dilutions:
- 1:200 (serum:dilution buffer),
- 1:100,
- 1:10,
- 1:1,
- undiluted serum.

On this basis, we estimated that the dilutions for our experiment should be between 1:100 and 1:10. Higher concentrations led to results exceeding the absorbance of the maximum standard concentration. Lower concentrations resulted in absorbance similar in all probes, what could hinder stating comparisons.

We then conducted another trials with dilutions as below:
- 3:100 - this concentration was finally chosen,
- 5:100.

For the IL-33 ELISA test no dilutions were needed, the test was performed with the non-diluted serum samples. Procedures were performed in accordance to the manual provided by the producer with ELISA kit (https://www.biorbyt.com/pub/media/additional_documents/o/r/orb390944.pdf).
